# Proximity matrix indicates heterogeneity in the ability to face child malnutrition and pandemics in Brazil: An ecological study

**DOI:** 10.3389/fpubh.2022.1019300

**Published:** 2022-11-10

**Authors:** Camila Botelho Miguel, Arianny Lima da Silva, Carlos Antônio Trindade-da-Silva, Melissa Carvalho Martins de Abreu, Carlo José Freire Oliveira, Wellington Francisco Rodrigues

**Affiliations:** ^1^Biosciences Unit, Medicine Course, University Center of Mineiros (UNIFIMES), Mineiros, GO, Brazil; ^2^Postgraduate Program in Tropical Medicine and Infectious Diseases, Federal University of Triângulo Mineiro (UFTM), Uberaba, MG, Brazil; ^3^Institute and Research Center São Leopoldo Mandic, São Leopoldo Mandic Faculty-SLMANDIC, Campinas, SP, Brazil

**Keywords:** social inequality, child malnutrition, COVID-19, mortality, Brazil

## Abstract

**Background:**

Among the social inequalities that continue to still surpasses the basic rights of several citizens, political and environmental organizations decisively “drag” the “ghost” of hunger between different countries of the world, including Brazil. The COVID-19 pandemic has increased the difficulties encountered in fighting poverty, which has led Brazil to a worrying situation regarding its fragility in the fight against new pandemics.

**Objectives:**

The present study aims to estimate, compare, and report the prevalence of mortality due to child malnutrition among the macro-regions of Brazil and verify possible associations with the outcome of death by COVID-19. This would identify the most fragile macro-regions in the country with the greatest need for care and investments.

**Methods:**

The prevalence of mortality was determined using data from the federal government database (DataSus). Child malnutrition was evaluated for the period from 1996 to 2017 and COVID-19 was evaluated from February to December 2020. The (dis)similarity between deaths from malnutrition and COVID-19 was evaluated by proximity matrix.

**Results:**

The North and Northeast regions have above average number of deaths than expected for Brazil (*p* < 0.05). A prospective analysis reveals that the distribution of the North and Northeast macro-regions exceeds the upper limit of the CI in Brazil for up to the year 2024 (*p* < 0.05). The proximity matrix demonstrated the close relationship between deaths from COVID-19 and malnutrition for the Northern region followed by the Northeast region.

**Conclusions:**

There are discrepancies in frequencies between macro-regions. Prospective data indicate serious problems for the North and Northeast regions for the coming years. Therefore, strategies to contain the outcome of health hazards must be intensified in the macro-regions North and Northeast of the country.

## Background

The coronavirus disease 2019 (COVID-19), which emerged in China, has put the world in a state of public calamity. Due to the severe acute respiratory syndrome caused by the SARS-CoV-2 virus, its morbidity and mortality rate, drastic measures like social isolation were recommended as the primary way to prevent the spread of the virus. During the critical period of the pandemic, restrictive measures were put in place regarding the operation of shops, holding sporting and religious events, closing schools, universities, and companies, in addition to containing borders and limiting foreign trade (1). Shortly after the critical period of the COVID-19 pandemic, the world encountered a new challenge in the form of food and energy insecurity due to the Russia–Ukraine war (2, 3).

However, regardless of the restrictive measures and the new global order, problems such as economic crisis, hunger, and misery have been increasing in several countries worldwide, including Brazil (4, 5).

According to previous estimates by the United Nations World Food Program, hunger indices worldwide will double as a result of COVID-19. The reasons include unemployment, decreased food consumption by closed establishments, crises in agricultural production, transport and export limitations (6). Currently, these concerns are being associated with armed conflicts and the insecurity of potential new pandemics (7).

In addition, measures like quarantine, social distancing and, more recently, the economic and social aggravations, have brought social inequalities into focus, especially considering the reduction in the purchasing power of the minimum wage and the inflation evidenced in several countries (8–10).

Regarding Brazil, a middle-income country, hunger has increased significantly after the pandemic. These impacts are exacerbated by the intense social inequality observed in the country (11). Some regions of the country, such as the states in the Northeast macro-region, experience greater socioeconomic problems. Thus, generating indicators that allow pointing out the most vulnerable macro-regions to factors linked to hunger, malnutrition and related to health problems, including pandemics, becomes important to allow the generation of indicators that contribute to optimizing prevention measures to combat social vulnerability.

Thus, the objective of this study was to evaluate the retrospective and prospective distributions of infant mortality due to malnutrition and the relationship between mortality rates and the aggravations of the COVID-19 pandemic in Brazil and its macro-regions.

## Materials and methods

### Study design and type

An ecological observational study was carried out over a period of 22 years (1996 to 2017) for data on deaths from child malnutrition (data recorded each year), and in the year 2020 for cases of deaths from COVID-19 (data recorded daily). The Ministry of Health database (Datasus) was consulted to obtain the data. Data on mortality from severe protein-calorie malnutrition and COVID-19 were considered for this study.

### Inclusion and exclusion criteria

Mortality data from severe protein-calorie malnutrition (ICD-10: E43) and from COVID-19 were included. The population of the different Brazilian macro-regions–North, Northeast, Southeast, South and Midwest–available from the database of the Ministry of Health, were evaluated. Data not validated by the Ministry of Health were not considered for this evaluation.

### Data extraction

The DataSus collection *via* Tabnet, made available by the Ministry of Health on its access page (http://tabnet.datasus.gov.br), was used to obtain data on infant deaths due to malnutrition. As for the deaths caused by COVID-19, the database of the Ministry of Health was accessed through the website: https://covid.saude.gov.br. The bases were accessed between March 1st and May 25th, 2022. To obtain population data and geographic coordinates were obtained from the database of the Brazilian Institute of Geography and Statistics (IBGE).

### Data processing and analysis

After accessing, the data were tabulated using the Excel program (Microsoft^®^). Statistical analysis was performed using Graphpad's SPSS 22.0 and Prism programs. Distribution (Kolmogorov-Smirnov with Dallal-Wilkinson-Liliefor *P*-value and Shapiro-Wilk) and variance (F test or Bartlett) were tested for all variables. Non-parametric tests were applied for comparison between groups (Mann-Whitney test and Kruskal-Wallis test), and data correlation (Spearman test). The annual mortality rates from child malnutrition between the period from 1996 to 2017 were used to optimize the linear regression model used to estimate the rates between the period from 2020 to 2025. The Euclidean distance between the variables was used to verify the (dis)similarities. To obtain the risk coefficient between the associations of mortality rates due to COVID-19 and malnutrition (shown on the map), the relative frequencies were initially determined for each variable (death from child malnutrition and COVID-19). To determine the frequencies of deaths from child malnutrition, the annual rates were added for each macro-region and then the total value (100%) was obtained by adding the frequencies for the five macro-regions, thus, the fractions for each macro-region were determined. To determine the relative frequencies of COVID-19 death rates, the same equation described above was used, however the sum for each macro-region was determined by the sum of the daily death rates. Thus, it was possible to obtain the ratio between the relative frequencies. QGIS software version 3.22.9 was used for geospatial optimization. The differences observed were considered significant when *p* < 0.05 (5%) (12).

## Results

Initially, the temporal impact on the prevalence of mortality from child malnutrition in the different macro-regions during the study period (1996 to 2017) was evaluated. A total of 3,895 deaths were recorded in 22 years, distributed across different macro-regions: North (*N* = 814; 20.90%), Northeast (*N* = 2005; 51.48%), Southeast (*N* = 585; 15.02%), South (*N* = 282; 7.24%) and Midwest (*N* = 209; 5.37%). After normalizing the data by population number, per 100,000 inhabitants, the means for the period evaluated were: 0.2542 ± 0.11 (North), 0.1804 ± 0.14 (Northeast), 0.03512 ± 0.03 (Southeast), 0.04865 ± 0.05 (South) and 0.0738 ± 0.06 (Midwest).

**Figure 1:**
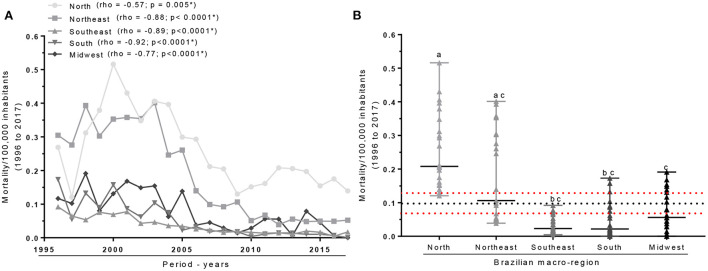
Correlation and comparison of mean distributions of the prevalence of mortality from severe child malnutrition from 1996 to 2017 for the Brazilian macro-regions. **(A)** Temporal correlation between mortality per 100,000 inhabitants and the period of 22 years. **(B)** Comparison between the frequencies of mortality per 100,000 inhabitants for the different macro-regions. Spearman's test was used to evaluate correlations and the Kruskal-Wallis test with Dunn's multiple comparison post-test was used to compare distributions (median with range). Statistically significant differences were considered when *p* < 0.05. The * and the letters a, b, and c indicate statistically significant differences.

The correlations between the prevalence of mortality per 100,000 inhabitants of the different macro-regions and the study period revealed negative and significant correlations (*p* < 0.05) for all the macro-regions (Figure 1A). The assessments of the distributions between the groups showed a heterogeneity of prevalence, pointing to high frequencies for the North and Northeast regions, followed by the Midwest, South, and Southeast regions. Both macro-regions, North and Northeast exceeded the line that shows the average distribution for Brazil in the evaluation period (0.018 ± 0.053 / 100 thousand inhabitants), which resulted in statistically significant differences between regions, *p* < 0.05 (Figure 1B).

After observing discrepancies in the distributions of mortality prevalence, as well as expressive correlations (*r*^2^ > 0.80), linear regressions were used to predict prevalence for the period from 2020 to 2025.

**Figure 2:**
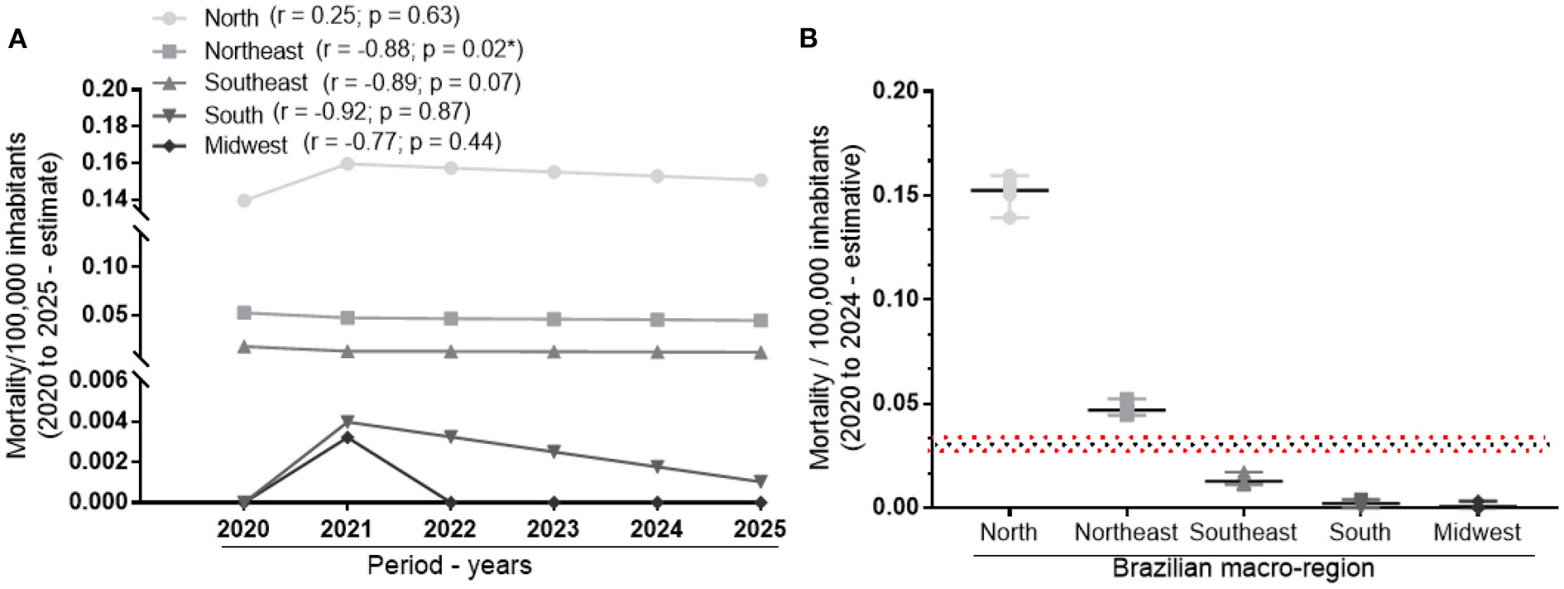
Prospective evaluation for correlation and comparison of mean distributions of the prevalence of mortality from severe child malnutrition between 2020 and 2025 for the Brazilian macro-regions. **(A)** Temporal correlation between mortality per 100,000 inhabitants and the period of six years. **(B)** Comparison between the average frequencies of mortality per 100,000 inhabitants for the different macro-regions. Spearman's test was used to evaluate correlations and the Kruskal-Wallis test with Dunn's multiple comparison post-test was used to compare distributions. Statistically significant differences were considered when *p* < 0.05. The * indicate statistically significant differences.

Next, regions were compared by estimating deaths from severe malnutrition (2020 to 2025), and the cutoff of the mean and confidence interval for Brazil for estimation was inserted. The data revealed a positive correlation of 0.25, although not significant (*p* = 0.63) but with a different profile from recent years for the Northern region, previously negative correlation. The other macro-regions continued with negative correlations, but only for the Northeast region it was statistically significant (*p* < 0.02) (Figure 2A). In the comparisons of the average distributions of the prospective evaluation, two of the five macro-regions (North and Northeast) were completely above the confidence interval for the average distribution expected for Brazil; and the others followed below the confidence interval line (Figure 2B).

**Figure 3:**
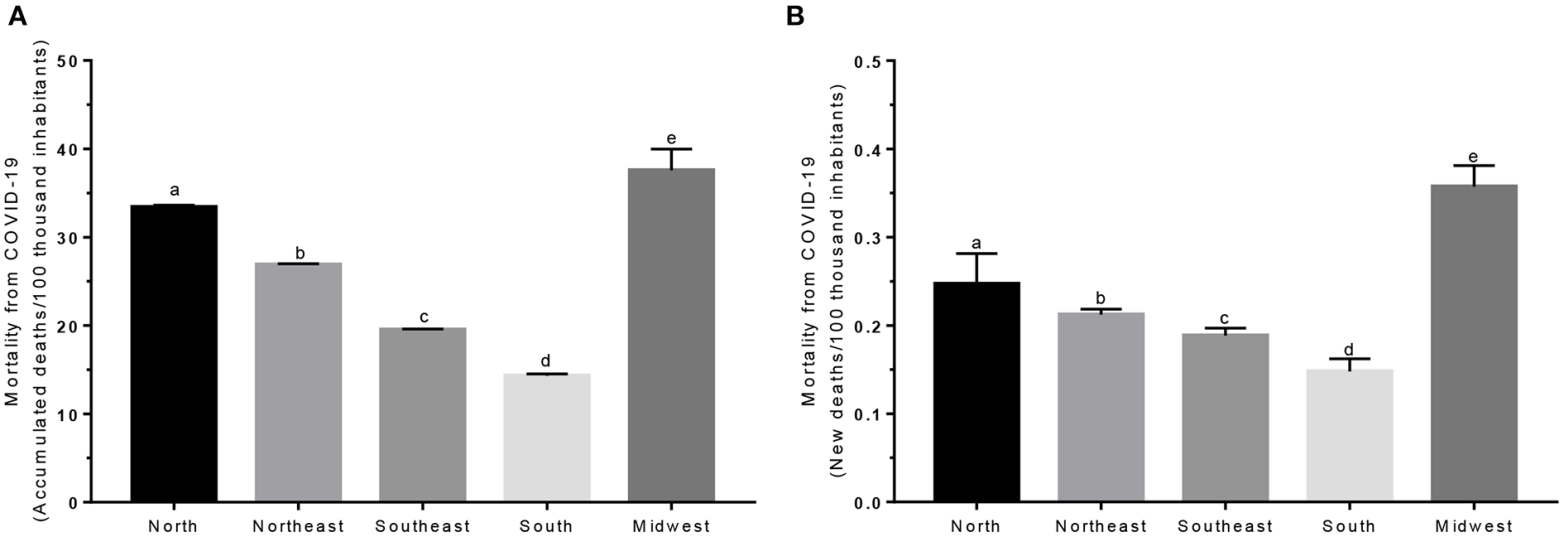
Comparison evaluation of the average distributions of mortality from COVID-19 in Brazil for the year 2020. Data were previously collected from the digital collection and made available by the Ministry of Health, for the year 2020. **(A)** Comparison of the mortality accumulated per 100,000 inhabitants, by COVID-19 to the different macro-regions. **(B)** Comparison of the mortality of new cases per 100,000 inhabitants, due to COVID-19 in the different macro-regions. The Kruskal-Wallis test with Dunn's multiple comparison post-test was used to compare the distributions. The letters a, b, c, d, and e demonstrate statistically significant differences, for *p* < 0.05.

Prior to the investigation of the possible relationships between deaths from child malnutrition and COVID-19, average comparisons were determined between the mortality per 100,000 for the different macro-regions, with regard to the accumulated death rate and new deaths from the beginning of notifications in Brazil until the initial date of December 2020. Comparisons showed significant differences between macro-regions (*p* < 0.05): 33.42 ± 37.26 (North), 26.90 ± 32 76 (Northeast), 19.53 ± 29.92 (Southeast), 14.31 ± 21.30 (South) and 37.55 ± 41.58 (Midwest). Thus, for both comparisons, cumulative mortality and new cases followed in descending order, the Midwest, North, Northeast, Southeast, and South regions (*p* < 0.05) (Figures 3A,B).

**Figure 4:**
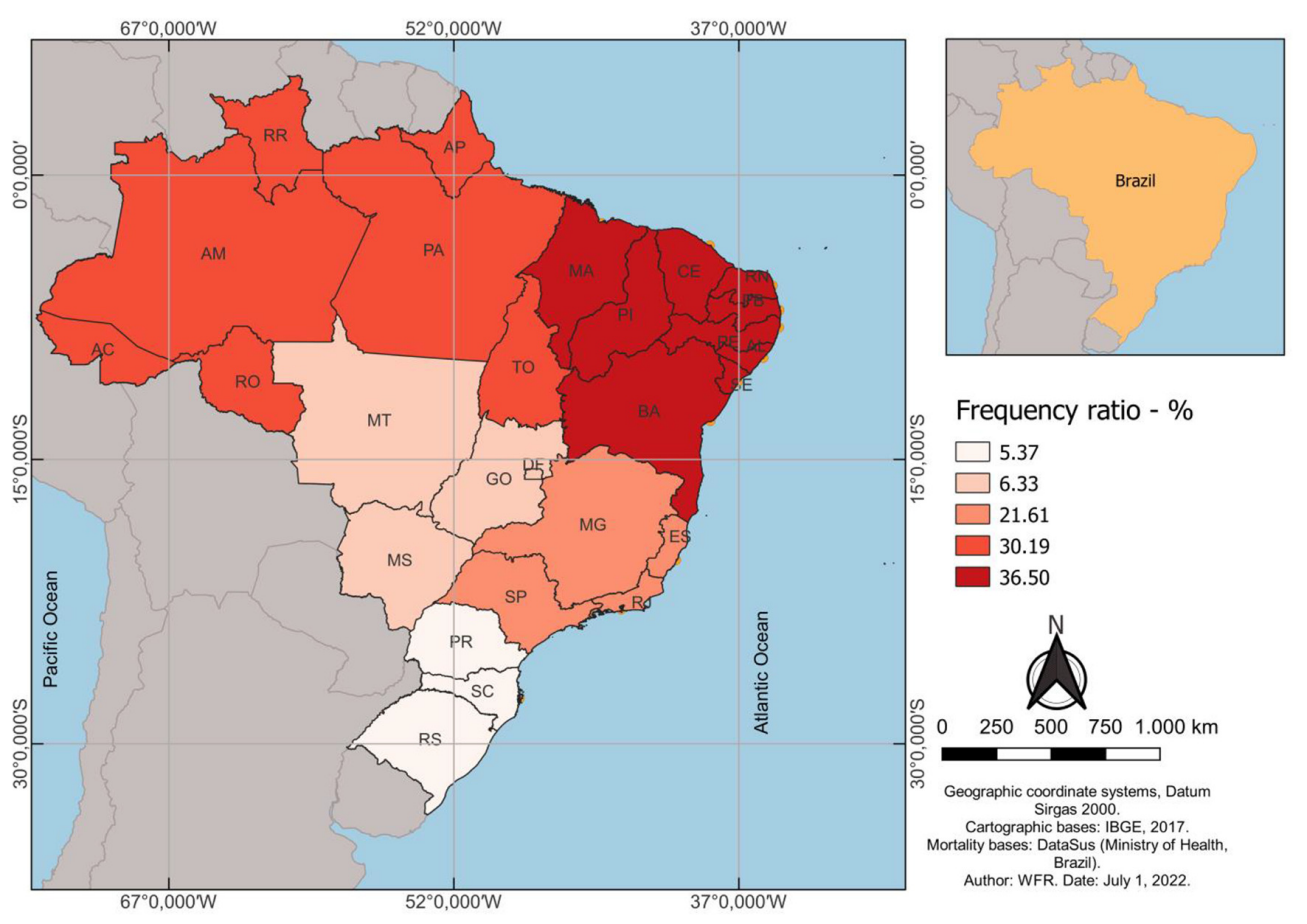
Distribution of risk frequencies for the association of the pandemic by COVID-19 and mortality rates due to malnutrition in Brazil. After obtaining the mortality rates from COVID-19 and malnutrition, the relative frequencies (%) of the rates for each macro-region of Brazil (north, northeast, southeast, south and midwest. The arithmetic mean between the relative frequencies of each macro-region was used to generate the risk frequency estimator and plotted for the federative units of each macro-region. On the map, the more intense colors show regions with greater relationships between mortality rates. The database of the Brazilian Institute of Geography and Statistics (IBGE) was consulted to obtain cartographic data. The coordinate system used was the Datum Sirgas 2000.

The effect size between the prevalence of mortality from COVID-19 and child malnutrition in Brazil was evaluated after checking the averages between the relative frequencies of mortality rates for each Brazilian macro-region (Figure 4). It was possible to identify an inequality between the macro-regions. The northeast region showed the greatest association in relation to the other macro-regions (36.50%), followed by the northern region of the country (30.19). The southern region was the macro-region with the lowest association (5.37) (Figure 4).

**Figure 5:**
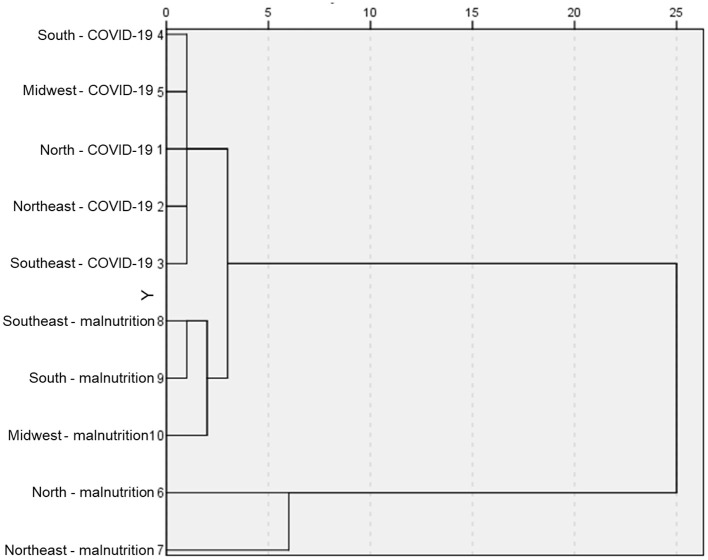
Analysis of (dis) similarity between the prevalence of mortality from malnutrition and COVID-19, in the different macro-regions of Brazil. Data were previously collecte'd from a digital collection and made available by the Ministry of Health. The (dis)similarity matrix was determined after evaluating the Euclidean distance. The dendrogram was used to assess the results.

To assess the regions critical to the impact of the association of mortality from malnutrition and COVID-19, a matrix of (dis) similarity was created, with subsequent plotting on a dendrogram (Figure 5). The evaluation revealed a link between the prevalence of mortality from malnutrition in the North and Northeast regions and the impacts of deaths from COVID-19, followed by the Southeast, South, and Midwest regions.

## Discussion

The evaluation of data related to mortality from child malnutrition per 100,000 inhabitants in Brazil, between 1996 and 2017, allows us to observe a negative correlation of the numbers over time. That is, there was a decrease in infant deaths from protein-calorie malnutrition in all macro-regions of Brazil. Compared to the global behavior of numbers related to the prevalence of child malnutrition over time, Brazil demonstrates that it follows this reality (13). In general, child malnutrition has shown a significant decrease worldwide. However, it remains a serious public health problem, especially in developing countries, as it transcends from an individual patient issue to a reflection of the society as a whole (14).

The last decades observed a decrease in the harmful conditions associated with the effects linked to malnutrition, particularly, infant mortality from malnutrition. However, relevant indices still persist and differ according to the macro-region analyzed. Although efforts have been made to reduce the damage linked to child malnutrition, despite the heterogeneity between the macro-regions, given their different social, cultural and geographical characteristics, the discrepancy in the outcome of the disease among children from different regions can be observed (15).

By studying the behavior of each macro-region individually, it was possible to trace the average of infant deaths due to protein-calorie malnutrition between the years mentioned, in each one of them, in addition to the Brazilian average in the same period of time. Thus, the North and Northeast regions stood out with the average mortality above the upper confidence interval of the general average for the country, as well as higher numbers when compared mainly to the Southeast region. This reality reflects the conditions of social inequality in Brazil, marked by intense economic and development differences between the extreme North and South of the country (16).

In the recent Brazilian literature, social inequality has been studied in the context of mortality from COVID-19. A greater increase in cases of COVID-19 fatality was observed in the states of Ceará, Pará, and Amazonas, which is in line with the reality of mortality from child malnutrition and also more prevalent in the states of the Northeast and Northern regions (17). One of the hypotheses that explains this reality is the limited access to health by the lower-income population, especially when it comes to primary care. For example, the annual average of medical consultations in the North of the country was almost half the average in the Midwest region in 2015 (15). Additionally, the beds of intensive care units available in the public network are significantly smaller than in the private network, making macro-regions unequal and poorer, the main challenge in the control of infections that generate pandemics.

Following this perspective, when projecting mortality values from child malnutrition for up to the year 2024, based on the linear regression of previous data, it was observed that the Northern region showed a positive correlation; that is, there are no positive data regarding to a possible drop in infant death rates due to malnutrition for the next few years in this region and no indicators of worsening numbers, since the correlation is not statistically significant. A possible association with this reality can be made from the justification of inequality between the states of the region, since the proportion of health impairment of populations residing in unequal regions is greater when compared to places that follow a socioeconomic homogeneity (18).

Conversely, following the projection analysis, the other macro-regions represented by the Southeast, South, Northeast, and Midwest showed a negative correlation. The Northeast region showed a statistically significant negative prospect. However, it still ranks second in the list of regions with the highest rates of mortality from malnutrition. It is also worth mentioning that this state holds 27% of the entire Brazilian population (16) and despite being very populous, it managed to remain below the leading infant death numbers. This is mainly due to policies to improve access to education for mothers, increase in family purchasing power, access to health, and basic sanitation (19).

Furthermore, when comparing the data projections for 2020 to 2024, the average and distribution of the estimate are above the expected level for Brazil in relation to the North and Northeast, with a greater focus on the Northern region. In the coming years, despite efforts to reverse the worrying situation regarding malnutrition in the Northeast, these macro-regions will continue to witness infant death from protein-calorie malnutrition in 2024.

When considering the current pandemic situation, these surveys become more alarming. Aspects such as poverty and unemployment, which are directly related to child malnutrition, have been identified as determinants of the incidence of numerous infectious diseases, including COVID-19, in Brazil (16).

With the setback of COVID-19, the differences with regard to the socioeconomic conditions of the Brazilian regions has become more evident (20). Poorer regions have experienced more severe effects of the pandemic. This is the case for the North and Northeastern regions, especially in relation to limitations of access to formal work, health and education (16).

According to a study released on the potential impacts of the pandemic, social isolation will affect all forms of malnutrition, especially in children. Analyzing the future projections, compromised child development, education, and deficit in the formation of human capital will be the challenges of the coming decades (21).

The destabilization of the international financial market and the lack of data associated with the indexes that are predictors of inflammation, along with the lack of updated and consistent data for hunger rates in the country were the main limitations of the study. In addition, the limitations associated with the design for the ecological study, with data originating from aggregates of information, as well as the use of secondary databases, can weaken individual indicators (22).

## Conclusions

Based on the retrospective and prospective analysis of child mortality data from malnutrition in Brazil, in the context of the COVID-19 pandemic, the North and Northeast regions tend to have greater negative impacts on child malnutrition due to the projections of the pandemic. These regions are arguably regions with greater vulnerabilities to face future pandemics or aggravations associated with low food demand or higher market inflation rates. It is, therefore, essential that measures be taken at the global, federal, state, and municipal levels to reverse the damage caused by the current humanitarian crisis.

## Data availability statement

The datasets presented in this study can be found in online repositories. The names of the repository/repositories and accession number(s) can be found below: the datasets generated and/or analyzed during the current study are available in the OSF Home repository, https://osf.io/ndc52/?view_only=547f494f5bbe4cea9f5f47b0041d3b5.

## Author contributions

WR, CM, CO, and AS designed the experiments. WR, CM, CO, AS, CT-d-S, and MA performed the experiments and wrote the manuscript. WR, CM, CO, and CT-d-S analyzed the data. All authors contributed to the article and approved the submitted version.

## Funding

This work was supported by the Federal University of Triângulo Mineiro (UFTM), University Center of Mineiros (UNIFIMES), and National Council for Scientific and Technological Development (CNPq; process: 152889/2022-1).

## Conflict of interest

The authors declare that the research was conducted in the absence of any commercial or financial relationships that could be construed as a potential conflict of interest.

## Publisher's note

All claims expressed in this article are solely those of the authors and do not necessarily represent those of their affiliated organizations, or those of the publisher, the editors and the reviewers. Any product that may be evaluated in this article, or claim that may be made by its manufacturer, is not guaranteed or endorsed by the publisher.
